# First Report of Two Cases of Acute Gastric Ischemia after Robot-Assisted Radical Cystectomy

**DOI:** 10.1155/2021/6697689

**Published:** 2021-01-27

**Authors:** Nikolaos Grivas, Alexander D. Horsch, Esther Wit, Annemarie Bruining, Johanna van Sandick, Henk G. van der Poel

**Affiliations:** ^1^Department of Urology, The Netherlands Cancer Institute-Antoni van Leeuwenhoek Hospital, Amsterdam, Netherlands; ^2^Department of Radiology, The Netherlands Cancer Institute-Antoni van Leeuwenhoek Hospital, Amsterdam, Netherlands; ^3^Department of Surgery, The Netherlands Cancer Institute-Antoni van Leeuwenhoek Hospital, Amsterdam, Netherlands

## Abstract

Gastrointestinal ischemia is rare after small pelvis surgery. Minimal invasive robotic surgery requires adaptation of the surgical approach for cystectomy and derivation construction such as the use of pneumoperitoneum and Trendelenburg positioning of the patient. Two cases with gastric ischemic complications after robot-assisted radical cystectomy are described. The first case was a 68-year-old female who had prolonged gastroparalysis and blood in a replaced gastric tube at day 10 after robotic cystectomy and Bricker urinary derivation. Gastroscopy revealed ischemia of gastric and proximal duodenal mucosa while computed tomography showed multiple calcifications and thrombi in the coeliac trunk branches and splenic infarcts. The stenosis of the origin of the mesenteric superior artery was stented via an endovascular procedure, and the patient recovered with normal gastroscopy 1 month postoperatively. The second case was a 73-year-old male who developed abdominal pain and fever 5 days after robotic cystectomy and Bricker. On abdominal computed tomography imaging, subcutaneous emphysema, intra-abdominal air, and calcification at the origin of the coeliac trunk were found. At laparotomy 5 days after the cystectomy, a 3 cm hole in the fundus of the stomach was found which was removed with the major stomach curvature. Gastroscopy 5 days after hemigastrectomy revealed no remnant ischemia. The prolonged pneumoperitoneum during robotic cystectomy, the deep Trendelenburg position, and the preoperatively impaired vascular system can be the reasons of our first two cases of gastric ischemia. This rare complication should be kept in mind in patients with symptoms of gastric ischemia since it can result in gastric perforation.

## 1. Introduction

Radical cystectomy is the gold standard for the treatment of muscle-invasive bladder cancer and high-risk nonmuscle invasive disease. Despite the widespread application and the increasing surgical experience with robot-assisted radical cystectomy (RARC), Clavien − Dindo ≥ 2 complications have been reported in 35-40% of the patients treated with RARC [1]. Ischemic complications can occur due to the increased intra-abdominal pressure associated with pneumoperitoneum applied during laparoscopic and robotic procedures, which can impair the splanchnic perfusion. Pneumoperitoneum itself and ischemia-reperfusion, after deflation of pneumoperitoneum, have been also found to decrease gastric pH and increase oxidative stress in patients undergoing robot-assisted radical prostatectomy with a prolonged duration of 4 hours [2]. Here, we present 2 cases with stomach ischemia after RARC both resulting in serious complications.

## 2. Case Presentation

### 2.1. Case 1

**Figure 1:**
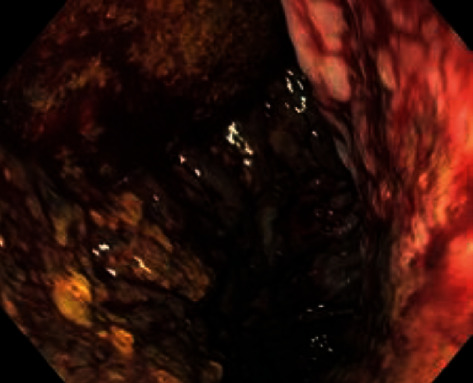
Gastroscopy 10 days postoperatively with gastric mucosal ischemia (case 1).

**Figure 2:**
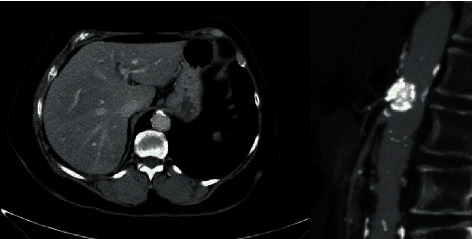
CT lateral view shows a severely calcified origin of the coeliac trunk and significant stenosis in the origin of the superior mesenteric artery. Axial image shows a thrombus inside the calcification (case 1).

A 68-year-old female with a prior history of multiple sclerosis, peripheral vascular atherosclerosis with an arterial stent in the left external iliac artery developed a multifocal nonmuscle invasive high-grade bladder cancer. Due to a low compliance bladder, multifocal disease, and suspected/potential muscle-invasive disease, it was decided to perform an anterior exenteration with the removal of bladder, urethra, uterus, and adnexes using a robot-assisted surgical approach. The procedure took 4 hours and included the construction of an intracorporeal Bricker urinary derivation. During the procedure, no bowel perfusion abnormalities were noted by the surgeons, but a remarkable short small-bowel mesentery was reported. Postoperative recovery was characterized by prolonged gastroparalysis and blood in a replaced gastric tube at day 10 postoperatively. Gastroscopy revealed ischemia of gastric and proximal duodenal mucosa (Figure 1). The Bricker stoma remained well perfused. Angio-computed tomography (CT) scan showed multiple calcifications and thrombi in the coeliac trunk branches and splenic infarcts (Figure 2). Stenosis of the origin of the mesenteric superior artery was stented (5 × 19 mm stent) via an endovascular procedure. One month after stent placement, there was a subsequent recovery with the improvement of clinical and gastroscopy findings. In the cystectomy specimen, a pTcisN0MxG3R0 urothelial carcinoma was found and the patient is recurrence-free three years after surgery.

### 2.2. Case 2

**Figure 3:**
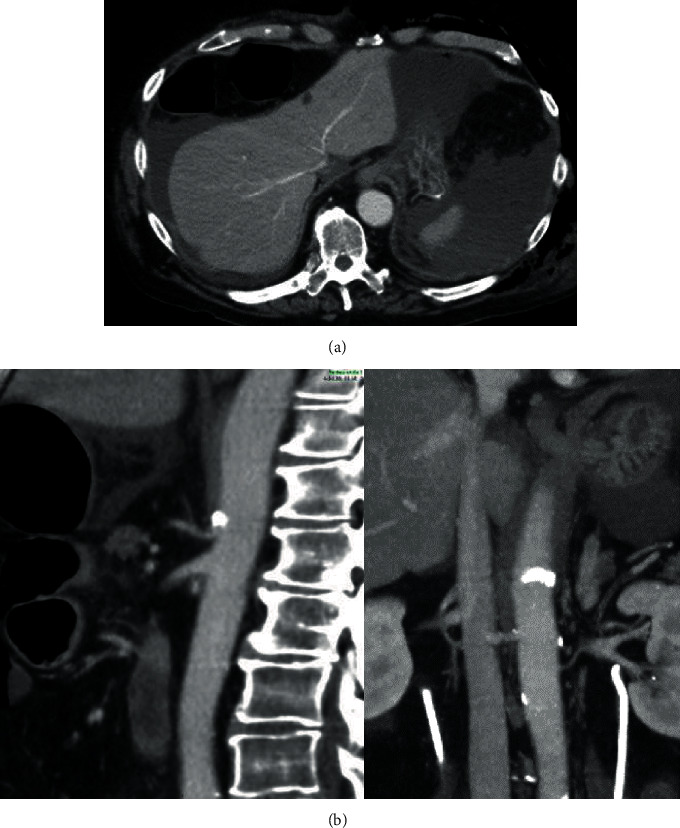
(a) CT with portovenous contrast shows intra-abdominal fluid around the stomach, spleen, and liver with free air suggestive of perforation (case 2). (b) CT lateral and axial views show a small-caliber coeliac trunk with calcification at the origin (case 2).

A 73-year-old male was seen with BCG-resistant carcinoma in situ of the bladder. The patient was known with hypercholesterolaemia, hypertension, and pulmonary embolism but no significant vascular disease. Five years after the primary diagnoses, RARC and pelvic node dissection with urine derivation according to Bricker was performed. Five days postoperatively, the patient developed abdominal pain, fever, and elevated serum CRP. On abdominal CT imaging, subcutaneous emphysema and intra-abdominal air were visible with perigastric and subphrenic fluid and renal ischemia on the left side, as well as calcification at the origin of the coeliac trunk (Figures 3 and 3(b)). At laparotomy 5 days after the RARC, a 3 cm hole in the fundus of the stomach was found with apparent ischemia of the major curvature of the stomach. The major curvature containing the hole in the stomach was removed using a surgical stapler, and the abdominal cavity was rinsed. Gastroscopy 5 days after hemigastrectomy revealed no remnant ischemia. The patient suffered pneumonia resulting in prolonged intensive care and showed a delayed recovery. The pathology of the cystectomy specimen revealed a pT1N0MxR0G3 urothelial carcinoma and a pT2N0MxR0GS3+4 prostate carcinoma. The gastrectomy specimen showed a perforation due to ischemia. The patient was discharged from the hospital 2 months after cystectomy but suddenly died in a nursing home 3 months after surgery. The autopsy revealed aorta sclerosis at the coeliac truncus but no signs of acute stomach ischemia.

## 3. Discussion

To the best of our knowledge, these are the first reported cases of gastric ischemia following RARC. In patients treated with robot-assisted radical prostatectomy, Luo et al. have shown that gastric pH is decreased by prolonged (4 hours) pneumoperitoneum [2]. It has been also found that low pH levels persist after termination of the pneumoperitoneum indicating that impaired splanchnic circulation continues even after deflation [2]. Moreover, the concentrations of malondialdehyde, which are the most reliable and producible markers of oxidative stress, were significantly elevated [2]. Ischemia-reperfusion can result in impaired myocardial and cerebral function, breakdown of the gastrointestinal barrier, and systemic and inflammatory response syndrome. Experimental models have also shown the clinical relevance of pneumoperitoneum-induced oxidative stress, with splanchnic ischemia while intestinal ischemia has been described after laparoscopic procedures [3, 4]. Since the severity of pneumoperitoneum-induced oxidative stress is time-dependent, splanchnic perfusion can be seriously impaired in a long operation as RARC (>4 h).

Patient-related risk factors can also impair splanchnic perfusion. Specific risk factors include advanced age, diabetes, hepatic and renal impairment, atherosclerosis, low cardiac output, cardiac arrhythmias, and medications (such as diuretics and beta-blockers) which can reduce intestinal perfusion [4]. In our study, both patients had cardiovascular disease symptoms and CT scans showed signs of vascular atherosclerosis, which could have predisposed to gastric ischemia. Calcified atherosclerotic plaques at the level of the coeliac trunk level in the aorta were present in both patients prior to surgery.

Prolonged Trendelenburg position can be also associated with gastric ischemia since it has been reported to be the cause also of acute intestinal distress syndrome [5]. This syndrome can especially occur in patients with preexisting cardiac and respiratory diseases who are in a prolonged, high-pressure Trendelenburg position [5]. This situation can result in high central venous and pulmonary arterial pressures while the cardiac output is decreased. The combination of Trendelenburg position with high CO_2_ pneumoperitoneum and a long surgical procedure can impair cardiac and vascular function, which could result in decreased gastric perfusion.

Measures to prevent this rare complication can be an increased intraoperative hydration, application of the lowest insufflation pressure possible, intermittent abdomen decompression, and use of novel smoke evacuation systems which combine stable intrabdominal pressure with constant suction via a mechanism of valve and insufflation. It has been associated with reduced need of CO_2_ insufflation, absorption, and elimination. Immediate gastroscopy in patients with symptoms of gastric ischemia (abdominal pain, nausea, vomiting, gastrointestinal bleeding) will allow an early diagnosis and improved survival. Treatment should include vascular support, antibiotic therapy, and vigorous use of proton-pump inhibitors. Patients with signs of ischemic stomach perforation require abdominal exploration, endovascular reconstruction, and/or partial gastrectomy.

## 4. Conclusion

The prolonged pneumoperitoneum during RARC, the deep Trendelenburg position, and the preoperatively impaired vascular system can be the reasons of our first two cases of gastric ischemia. This rare complication should be kept in mind in patients with symptoms of gastric ischemia since it can result in gastric perforation.

## Data Availability

No data were used to support this study.

## Abbreviations

RARC: Robot-assisted radical cystectomy
CT: Computed tomography.
